# Omega-3 Fatty Acids from Fish Oil Lower Anxiety, Improve Cognitive Functions and Reduce Spontaneous Locomotor Activity in a Non-Human Primate

**DOI:** 10.1371/journal.pone.0020491

**Published:** 2011-06-07

**Authors:** Nina Vinot, Mélanie Jouin, Adrien Lhomme-Duchadeuil, Philippe Guesnet, Jean-Marc Alessandri, Fabienne Aujard, Fabien Pifferi

**Affiliations:** 1 Mécanismes Adaptatifs et Evolution, UMR 7179 Centre National de la Recherche Scientifique, Muséum National d'Histoire Naturelle, Brunoy, France; 2 Laboratoire de Nutrition et Régulation Lipidique des Fonctions Cérébrales, INRA de Jouy en Josas, Domaine de Vilvert, Jouy en Josas, France; Pontifical Catholic University of Rio Grande, Brazil

## Abstract

Omega-3 (ω3) polyunsaturated fatty acids (PUFA) are major components of brain cells membranes. ω3 PUFA-deficient rodents exhibit severe cognitive impairments (learning, memory) that have been linked to alteration of brain glucose utilization or to changes in neurotransmission processes. ω3 PUFA supplementation has been shown to lower anxiety and to improve several cognitive parameters in rodents, while very few data are available in primates. In humans, little is known about the association between anxiety and ω3 fatty acids supplementation and data are divergent about their impact on cognitive functions. Therefore, the development of nutritional studies in non-human primates is needed to disclose whether a long-term supplementation with long-chain ω3 PUFA has an impact on behavioural and cognitive parameters, differently or not from rodents. We address the hypothesis that ω3 PUFA supplementation could lower anxiety and improve cognitive performances of the Grey Mouse Lemur (*Microcebus murinus*), a nocturnal Malagasy prosimian primate. Adult male mouse lemurs were fed for 5 months on a control diet or on a diet supplemented with long-chain ω3 PUFA (n = 6 per group). Behavioural, cognitive and motor performances were measured using an open field test to evaluate anxiety, a circular platform test to evaluate reference spatial memory, a spontaneous locomotor activity monitoring and a sensory-motor test. ω3-supplemented animals exhibited lower anxiety level compared to control animals, what was accompanied by better performances in a reference spatial memory task (80% of successful trials *vs* 35% in controls, p<0.05), while the spontaneous locomotor activity was reduced by 31% in ω3-supplemented animals (p<0.001), a parameter that can be linked with lowered anxiety. The long-term dietary ω3 PUFA supplementation positively impacts on anxiety and cognitive performances in the adult mouse lemur. The supplementation of human food with ω3 fatty acids may represent a valuable dietary strategy to improve behavioural and cognitive functions.

## Introduction

The brain cells membranes of Vertebrates, including primates, have high concentrations of long-chain polyunsaturated fatty acids (PUFA) of the omega-3 (ω3) and omega-6 (ω6) series, mainly docosahexaenoic acid [DHA, 22∶6 (n-3)] and arachidonic acid [AA, 20∶4 (n-6)] [1]. The specific accretion of DHA (the main ω3 PUFA in brain cells membranes) during perinatal development is considered to be essential for the proper functioning of the mammalian central nervous system. The role of ω3 fatty acids has been mainly investigated using the dietary deficiency model in which animals, mainly rodents, are deprived of any source of ω3 fatty acids during the perinatal development (“chronic deficiency”). It has been demonstrated that decreased content of DHA is accompanied by severe neuro-sensorial impairments (learning, memory, anxiety) that have been linked to changes in neurotransmission processes [2]. Conversely to chronic deficiency, few studies investigated the impact of ω3 PUFA supplementation on behavioural and cognitive functions.

Several studies showed that chronic ω3 PUFA deficiency increased anxiety, particularly when animals were placed in an anxiogenic environment. ω3-deficient mice and rats exhibited significant signs of anxiety [3], [4] more particularly under stressful conditions [3]. Harauma and Moriguchi [5] recently confirmed that dietary ω3 PUFA deficiency in mice augments chronic mild stress-induced anxiety. Furthermore, supplementation with ω3 PUFA of mice and rats previously raised under an ω3 PUFA-deficient diet reduced their level of anxiety while restoring normal fatty acid composition in most brain regions [6], [4]. Confirming the impact of dietary ω3 PUFA on anxiety, the exploratory behaviour in a novel environment (anxiogenic) was reduced in ω3-deficient rats [7] and improved in ω3-supplemented mice [4]. These outcomes may be related to increased and lowered anxiety in deficient and supplemented animals, respectively.

In addition to its impact on anxiety, chronic ω3 PUFA deficiency has been shown to impair reference spatial memory in a circular maze (Barnes maze) in rats [8] and mice [9]. Furthermore, studies in rats [10], mice [11] and hamsters [12] demonstrated that ω3 PUFA deficiency led to hyperactivity, and more particularly to increased spontaneous locomotor activity. These observations are confirmed by a supplementation study in which rats raised on a fish oil diet exhibited reduced locomotor activity compared to controls [13].

Animal studies, more particularly those performed in rodents, clearly indicate that long chain ω3 fatty acids play a role in behavioural and cognitive functions. The impact of dietary ω3 fatty acids on brain functions in non-human primates has been studied in a much more small number of works. Tsukada et al. [14] demonstrated that supplementing aged monkeys with DHA for 1 to 4 weeks (a very short term dietary supplementation) led to increased regional cerebral blood flow, a parameter closely linked to neuronal activation. Since ω3 PUFA supplementation may be effective in the treatment of depressive disorder (reviewed in [15]) it was recently proposed that they may also possess anxiolytic properties [16]. In humans, few data are available from intervention studies about the association between anxiety and ω3 fatty acids supplementation and data are divergent about the ability of dietary ω3 fatty acids to prevent age-associated cognitive decline (see [17] for review). Therefore, the development of nutritional studies in non-human primates is needed to disclose whether a long-term supplementation with long-chain ω3 PUFA has an impact on behavioural and cognitive parameters, differently or not from rodents.

Mouse lemur (*Microcebus murinus*) is a nocturnal prosimian primate originating from Madagascar with a life expectancy of 8–10 years. The grey mouse lemur presents specific characteristics that make it a good model to evaluate the effects of long-term dietary treatments on behavioral and cognitive parameters in primates. In particular they present a small size and weight (80 to 120 g), omnivorous dietary habits, and the possibility to assess their behavioral and cognitive performances with specific tasks which have been developed and adapted in our laboratory [18], [19].

We compared the effects of a 5 months supplementation with long-chain ω3 fatty acids or with monounsaturated fatty acids (iso-caloric control diet), on anxiety, cognitive and motor functions in the adult mouse lemur. Anxiety was specifically assessed using the open field task. To evaluate cognitive performances, we used the circular platform task which recruits the hippocampal system-dependent spatial memory and which is known to be particularly adapted for the mouse lemur [19], [20]. No food reinforcement is required, thus motivation to achieve this task is independent of appetite. In addition, spontaneous locomotor activity in non anxiogenic environment was evaluated. Motor abilities were also assessed using the Rotarod test to make sure that differences in physical performances did not interfere with anxiety and cognitive performances.

In animal studies, dietary treatments with PUFA are mainly applied during the perinatal period of developpment (more particularly for studies on brain functions) and very few studies are performed with dietary interventions at adult age. There is a particular interest to determine whether dietary PUFA impact on brain functions at adult age, inasmuch as the mean dietary intakes of long chain ω3 PUFA in adults are below the levels of recommendation in developped countries [21]. In the present study we focused on young adult mouse lemur subjected to a level of intake corresponding to the recommendation for the French adult population [22].

The aim of the present study was to evaluate the impact of a long-term dietary supplementation with ω3 fatty acids (under the form of fish oil, naturally rich in long-chain ω3 fatty acids) on behavior, cognition and motor performances in adult primates. We postulated that fish oil supplementation may improve behavioral and cognitive parameters which could be of major importance in the perspective of their use as supplemental ingredient in human foods.

## Materials and Methods

### 1 Ethics Statement

All experiments were performed in accordance with the *Principles of Laboratory Animal Care* (National Institutes of Health publication 86–23, revised 1985) and the European Communities Council Directive (86/609/EEC). The Research was conducted under the authorization n° 91–305 from the “Direction Départementale des Services Vétérinaires de l'Essonne” and the Internal Review Board of the UMR 7179. All the experiments were done under personal license (authorization number 91–460, issued 5 June, 2009) delivered by the Ministry of Education and Science. In accordance with the recommendations of the Weatherall report, “The use of non-human primates in research”, special attention was paid to the welfare of animals during this work [23]. All efforts were made to minimize nociception.

### 2 Animals and diets

Adult male grey mouse lemurs (*Microcebus murinus*, Cheirogaleidae, primates) were handled during the summer-like long day length (14∶10 h light-darkness) that corresponds to the active phase of the animals. Twelve animals (6 per diet group) were included at the age of 23±4 months. Animals were raised on fresh fruits and a laboratory daily-made mixture of cereals, milk and egg. Water and food were given *ad libitum*. Animals were randomly assigned to each experimental group. The ω3-supplemented group received the home-made food supplemented with tuna oil, rich in long-chain ω3 PUFA, while the control group received the food supplemented for isoenergy with the same volume of olive oil (rich in monounsaturated fatty acids and poor in ω3 fatty acids). In the tuna oil supplemented group, the intakes of eicosapentaenoic acid (EPA, 20∶5 n-3) and of docosahexaenoic acid (DHA, 22∶6 n-3) represented about 0.06% and 0.3% of total energy, which is equivalent to the highest level of consumption of French coastal populations [24] and corresponds to the recommended daily intake for the French population [22]. These proportions correspond to a daily intake of 6 mg EPA and 30 mg DHA per animal. Cognitive and motor tests were performed between the fifth and the sixth month of supplementation. Body weights were measured all along the protocol and remained significantly unchanged with dietary treatments.

### 3 Lipid analysis

The blood was collected on heparin, centrifuged and the plasma was stored at −80°C until analysis. Total lipids were extracted from plasma with chloroform/methanol 2/1 using the method of Folch. Total plasma phospholipids were isolated by solid phase liquid chromatography on silica cartridges; sequential elution was made with chloroform, then with methanol, which contained the phospholipid fraction [25]. All eluents were dried under nitrogen, and the phospholipid fractions were transmethylated with 10% boron trifluoride (Fluka, Sokolab) at 90°C for 20 min [26]. Fatty acids methyl esters were analyzed by gas chromatograpy [27]; the fatty acids composition is expressed as a weight percentage (g/100 g of total fatty acids).

### 4 Circular platform task

The circular plastform task apparatus was an adaptation of the device described by Barnes [28] for mouse lemurs. It consisted of a white circular platform (diameter, 100 cm) with 12 equally spaced circular holes (each 5 cm in diameter) located 3 cm from the perimeter. The platform could be rotated. The maze platform was placed 60 cm above the floor, and a cardboard nestbox (10 cm×10 cm×20 cm) could be inserted and removed beneath each hole and served as a refuge (goal box). A black, small plywood box could be slid beneath the non-goal holes to stop the lemurs from jumping through these holes while permitting head entering. To prevent the mouse lemur from escaping, the platform was entirely surrounded with a white wall 25 cm high and covered with a transparent Plexiglas® ceiling that permitted the mouse lemurs to see the extra-maze visual cues. The apparatus was surrounded by a black curtain hung from a square metallic frame (length of the side, 120 cm) located 110 cm above the floor. The center of the frame was a one-way mirror to allow observation. Attached beneath the one-way mirror and along perimeter of the maze (about 50 cm above the platform) were 24 evenly spaced 2-W lights, illuminating the maze. Between the one-way mirror and the upper edge of the wall, various objects were attached along the inner surface of the curtain to serve as visual cues. The starting box was an open-ended dark cylinder positioned in the center of the platform. Transparent radial Plexiglas partitions (25 cm high×20 cm long) were placed between the holes to prevent the strategy used by some mouse lemurs to go directly to the periphery of the platform and then walk along the wall and inspect each hole one by one. Consequently, animals had to return to the center of the platform after each hole inspection.

#### Testing procedure

Animals were given one session of habituation and training (day 1) and one session of testing (day 2). Each session included four trials, each of which began with placement of the animal inside the starting box. After 30 seconds, the box was lifted to release the animal. For the lemurs, the objective was to reach the goal box positioned beneath one of the 12 holes, kept constant relative to the cues for all trials. When the animal entered the goal box, the trial was stopped, and the animal was allowed to remain in the goal box for 3 minutes. After each trial, the platform was cleaned and randomly rotated on its central axis to avoid the use of intra-maze cues, although the position of the goal box was kept constant relative to the cues.

On day 1, trials 1 and 2 consisted of placing the animal in a four-walled chamber containing only the opened goal hole (one-choice test). For trials 3 and 4, the platform comprised six evenly spaced open holes (six-choices test). These two trials permitted the animal to explore the maze, observe the visual cues, and further learn the position of the goal box.

On day 2 (testing day), 12 holes were opened during the four trials. Performance was assessed based on the time required for the animal to reach the right exit (expressed in sec) and the number of errors and visits prior to reaching the goal box. For each group, the rate of success was also defined as the ratio of successful trials on the total number of trials during the testing day, expressed in %.

### 5 Open field task

This system was an open-field consisting of bright and opaque Plexiglas® wall (100×100×20 cm) and covered with a transparent Plexiglas® ceiling. Four white lights of 15 W were placed at each corner of the system. The open field session was recorded by camera and the data were analyzed after the session, which rendered unnecessary the presence of an observer in the room during the test.

#### Testing procedure

The mouse lemurs were placed in the open-field for free exploration for 30 min. At the end of the session, the nestbox of the mouse lemur was placed in a corner of the open field (the same corner for all animals) to allow him to return to its nestbox with a minimal stress. Because of persistent immobility, peripheral tracking and limited exploration are index of stress and anxiety in mouse lemurs when placed in a novel environment. We determined three parameters reflecting the degree of anxiety for each animal: total distance travelled during the test (expressed in cm), activity duration during the test (expressed in s) and number crossings of the central zone.

### 6 Accelerating rotating rod task

This apparatus allowed quantification of fine motor coordination and balance by measuring the amount of time that a mouse lemur could remain standing on a rotating, accelerating rod (model 7750, Ugo Basile, Italy). The rod was a plastic drum, 5 cm in diameter, which was machined to provide traction. The rotational speed of the system could be progressively increased up to 40 rpm.

#### Testing procedure

The animal was placed on the rotating cylinder at 20 rpm. The rod then accelerated steadily until the end of the test which was reached when the animal fell or gripped onto the rod during at least three consecutive turns without stabilizing its balance. Latency to fall or grip on the rod was recorded for each trial. Animals underwent 5 consecutive trials and the best performance was recorded. Values were expressed in seconds.

### 7 Spontaneous locomotor activity

Animals were housed individually in a laboratory-made locomotor activity cage with a capacity of 1 m^3^ each provided with nestbox and supports. Spontaneous locomotor activity was estimated using a system of presence and motion sensors placed in the cage and the nestbox created by R. Botalla and adapted to the mouse lemur. Presence sensors (Honeywell – transmitter: SEP8705003, receiver: SDP8405014) were placed on both sides of the nest and were continuously recording in order to detect animal presence in the nest. Two motion sensors (GARDTEC – Gardscan ‘M’ series infra-red detectors) were placed in the corners of the cage to detect the spontaneous movements of the animal. During animal movements the motion sensors recorded data every two seconds. Data were stored in a computerized system (developed in the laboratory by R. Botalla). They were then computed to represent time-course of these movement patterns using a software filtering “ACTOCEBE 3.0” developed in language G from National Instruments (software created by R. Botalla). Based on animal activity, total movements were averaged over 5 minutes intervals for further analysis and were expressed in arbitrary unit (a.u.). A particular focus was given to night locomotor activity, which is the active period of Grey mouse lemurs. After 48 h of habituation, spontaneous locomotor activity was recorded during 5 consecutive days for each animal.

### 8 Statistical analyses

All values are expressed as mean ± SEM. Unpaired Student's T-test analyses were used to assert significant variations between the control group and the ω3-supplemented group in all studied parameters. Comparisons were considered to differ significantly with p<0.05. All statistical computations were performed with Prism 5 for Windows XP (Graphpad software).

## Results

### 1 Plasma lipids

Quantification of fatty acids in the plasma total phospholipids (Table 1) demonstrates that tuna oil supplementation significantly increased the level of ω3 fatty acids and compensatorily decreased that of ω6 and monounsaturated fatty acids. Tuna oil supplemented animals exhibited a 3-fold increase in ω3 fatty acids compared to controls (from 8.1±0.9% of total fatty acids in control to 24.7±1.1% in supplemented animals, p<0.01; t = 20.48, df = 10) while total ω6 and monounsaturated fatty acids concurrently decreased by 42% (p<0.01; t = 12.43, df = 10) and 31% (p<0.01; t = 22.05, df = 10) respectively. DHA was notably increased close by 160% (p<0.01; t = 14.72, df = 10) when AA was decreased by 87% (p<0.01; t = 9.637, df = 10) upon tuna oil supplementation. The ratio of total ω6∶total ω3 PUFA was equal to 0.83∶1 in tuna oil supplemented animals and to 4.35∶1 in olive oil supplemented group. The saturated fatty acids were not substantially altered by dietary treatment.

**Table 1:** Plasma fatty acids from total phospholipids of control and tuna oil supplemented animals.

	Control (*n* = 6)	Fish oil (*n* = 6)
Fatty acids^2^	g/100 g^1^	
12∶0	0.5±0.2	1.9±0.1
16∶0	24.8±0.6	27.2±0.3*
18∶0	16.7±0.8	14.4±0.2*
**Σ Saturated**	**43.3±0.0**	**45.0±0.6**
18∶1 n-9	9.0±0.0	6.0±0.2*
18∶1 n-7	1.8±0.0	1.3±0.0
**Σ Monounsaturated**	**11.6±0.0**	**8.0±0.3** *
18∶2 n-6	13.4±1.4	8.9±0.7*
20∶4 n-6^a^	16.3±0.1	9.6±1.2*
22∶4 n-6	1.5±0.3	0.2±0.1
22∶5 n-6	1.5±0.2	0.4±0.0
**Σ n-6 Polyunsaturated**	**35.3±0.8**	**20.5±1.9** *
18∶3 n-3	0.1±0.0	0.1±0.0
20∶3 n-3	0.0±0.0	0.0±0.0
20∶4 n-3	0.1±0.0	0.1±0.0
20∶5 n-3^b^	0.2±0.0	4.0±0.6*
22∶5 n-3	1.7±0.3	4.9±0.8*
22∶6 n-3^c^	6.0±0.7	15.5±0.9*
**Σ n-3 Polyunsaturated**	**8.1±0.9**	**24.7±1.1** *

1 Values are means ± SEM, n = 6.

*indicates significant differences between dietary treatments with p<0.01.

2 Minor fatty acids [14∶0, 15∶0, 17∶0, 19∶0, 20∶0, 22∶0, 24∶0, 14∶1(n-5), 16∶1(n-9), 20∶1(n-7), 20∶1(n-11), 22∶1(n-7), 24∶1(n-11), 24∶1(n-7), 20∶3(n-9), and 22∶3(n-9)] are not reported because they represented <0.3% of total fatty acids.

a 20∶4 n-6: Arachidonic Acid (AA),

b 20∶5 n-3: Eicosapentaenoic Acid (EPA),

c 22∶6 n-3: Docosahexaenoic Acid (DHA).

### 2 Open field task

**Figure 1:**
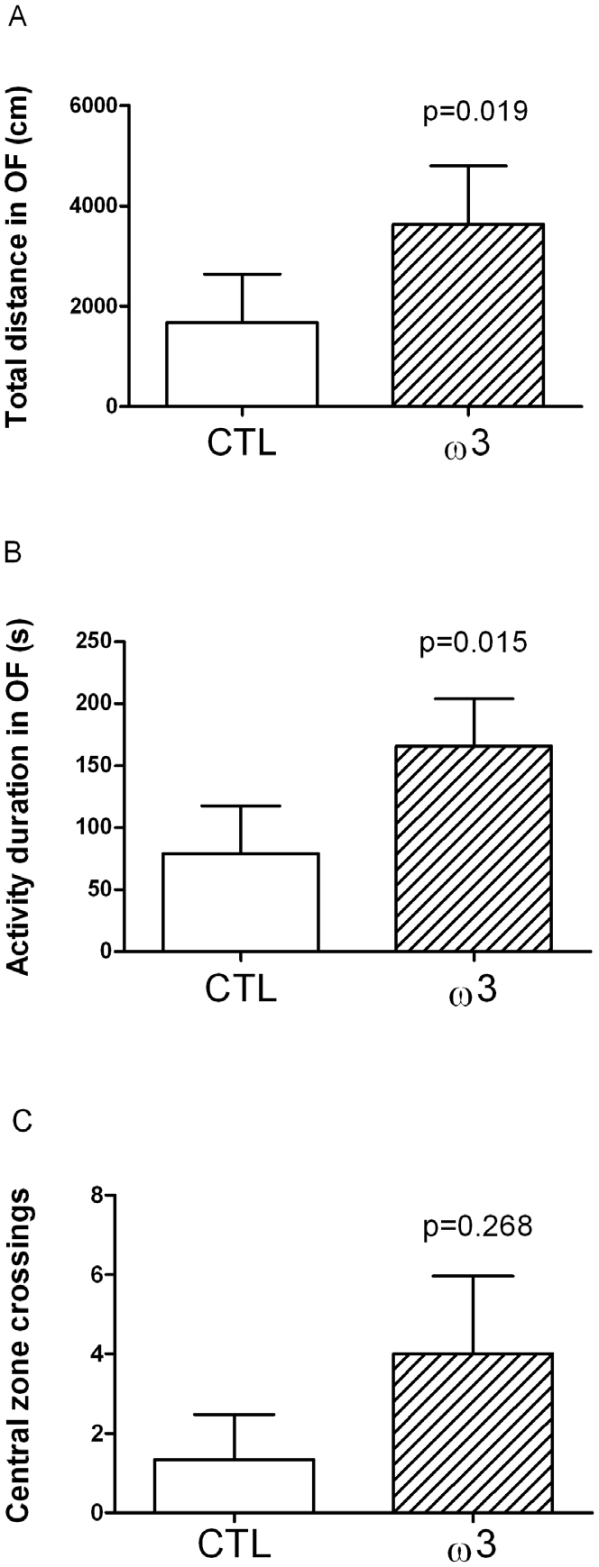
Performances in an open field (OF) task. A. Total distance travelled (cm). B. Activity duration (s). C. Number of times the animals crossed the central zone. Values are means ± SEM, n = 6 in each dietary group. Differences were considered significant between control (CTL) and tuna oil supplemented animals (ω3) with p<0.05.

The total distance travelled during the open field task was significantly longer (p = 0.019; t = 2.830 df = 10) in animals fed the tuna oil (3628±1170 cm) compared to controls (1674±967 cm) (Figure 1A) as was the activity duration (165.8±38.2 s for tuna oil supplemented animals and 79.2±38.4 s for control animals, p = 0.015; t = 2.916; df = 10) (Figure 1B). The number of times the animals crossed the central zone was not significantly different (p = 0.268; t = 1.172, df = 10) between tuna oil supplemented animals (4.0±2.0) and controls (1.3±1.1) (Figure 1C).

### 3 Circular platform task

**Figure 2:**
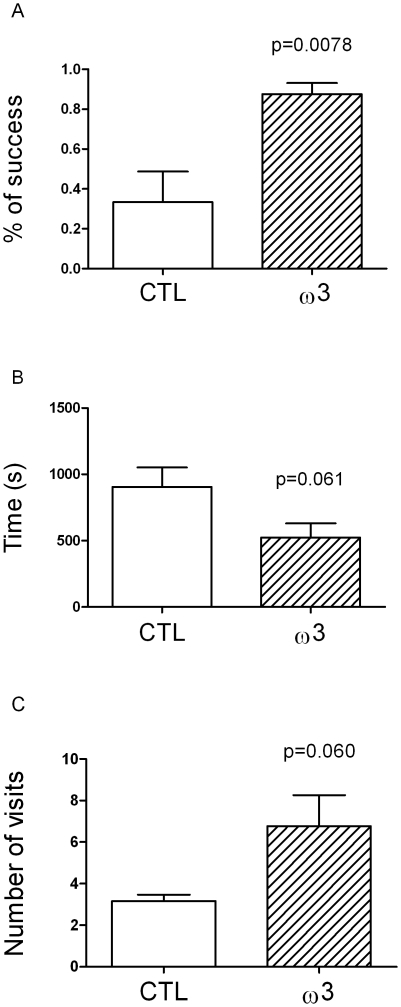
Performances in a circular maze. A. Rate of success, expressed as the ratio of successful trials on the total number of trials (%). B. Time before reaching the right exit (s). C. Total number of visits. Values are means ± SEM, n = 6 in each dietary group. Differences were considered significant between control (CTL) and tuna oil supplemented animals (ω3) with p<0.05.

The success rate in the circular platform task is expressed as the number of successful trials on the total number of trials during the testing day (in %, Figure 2A). Animals of the tuna oil group exhibited 87.5±5.0% of success in this task compared to 33.3±15.4% for animals of the control group (p = 0.0078, t = 3.313, df = 10). Moreover, the tuna oil supplemented animals tended to spend less time to exit from the maze compared to controls (524±106 s vs 906±145 s; p = 0.060, t = 1.706, df = 10) (Figure 2B), while their exploratory activity (total number of visits) was increased (6.8±1.5 vs 3.2±0.3; p = 0.061, t = 2.117, df = 10) (Figure 2C), although these differences did not reach the level of statistical significance.

### 4 Accelerating rotating rod task

**Figure 3:**
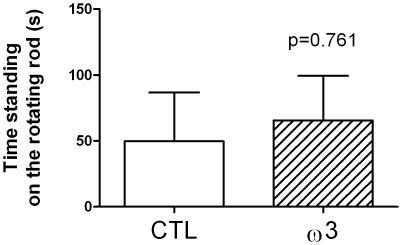
Performances in an accelerating rotating rod task. Data are expressed as the time standing on the rotating rod in s. Values are means ± SEM, n = 6 in each dietary group. Differences were considered significant between control (CTL) and tuna oil supplemented animals (ω3) with p<0.05.

No significant differences were observed for the performances in the accelerating rotating rod task (Figure 3) between tuna oil supplemented animals (65.5±34.0 s) and controls (49.8±37.0 s) in the time standing on the rotating rod (p = 0.761; t = 0.3116, df = 10).

### 5 Spontaneous locomotor activity

**Figure 4:**
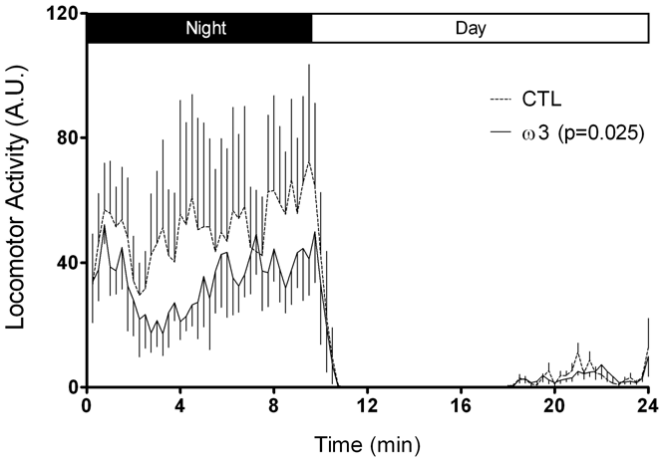
Spontaneous night and day locomotor activity. Data were recorded during 5 consecutive 24 h periods (expressed in arbitrary units of locomotor activity). Values are means ± SEM, n = 6 in each dietary group. Differences were considered significant between control (CTL) and tuna oil supplemented animals (ω3) with p<0.05.

Spontaneous night and day locomotor activity was expressed on the same scale in arbitrary units (figure 4). Tuna oil supplemented animals exhibited 31% less nocturnal locomotor activity compared to controls (p<0.001; t = 7.619; df = 76) whereas both groups had low and similar day locomotor activity (p = 0.673; t = 0.4230; df = 112).

## Discussion

We investigated the effects of a 5 month long-chain ω3 PUFA supplementation on behavioural, cognitive and locomotor performances in adult mouse lemurs. Our results evidence for the first time in a non-human primate species that ω3 PUFA supplementation lowered anxiety and spontaneous locomotor activity and concomitantly improved cognitive performances.

Plasma fatty acids from total phospholipids confirmed that animals receiving the tuna oil-supplemented diet had significantly higher levels of circulating long chain ω3 PUFA (including EPA, 22∶5n-3 and DHA) compared to controls. The brain fatty acid compositions being not accessible without the sacrifice of animals, plasma fatty acids constitute a proper marker of body fatty acids composition. Indeed, it has been demonstrated in several mammal species that an increase in dietary DHA correlates with plasma DHA content which is in turn predictive of internal organ DHA status and is also a useful biomarker of brain DHA status during adulthood [29]. The increased level of ω3 PUFA in tuna oil supplemented animals occurred at the expense of both ω6 PUFA and monounsaturated fatty acids. These changes contributed to improve the balance between ω3 and ω6 PUFA in the plasma phospholipids of tuna oil supplemented animals with a ratio of ω6∶ω3 of 0.83∶1 compared to 4.35∶1 in the olive oil group. It is recommended for human health benefits that the dietary ratio of ω6∶ω3 fatty acids should be close to 1∶1 [30].

ω3 PUFA-supplemented animals exhibited increased activity during the open field task suggesting a reduction of their level of anxiety. As suggested by Prut and Belzung [31], in this task, anxiolytic treatments (such as ω3 PUFA in our case) do not themselves increase exploration in the open field but decrease the stress-induced inhibition of exploration behaviour. However we did not observe significant changes in the number of crossings of the central zone, a parameter closely linked to anxiety, suggesting a moderate anxiolytic effect of long chain ω3 PUFA. This observation is corroborated by the lowered spontaneous locomotor activity in a familiar environment measured for ω3-supplemented animals. In this test, performed in their breeding room, animals are habituated during 48 h to their new cage (similar in size and form to their home-cage) previously to the recording of spontaneous locomotor activity during 5 consecutive days. In such conditions, locomotor activity is generally gradually reduced over the testing as the animals are habituated to the cage environment [3]. The observation of reduced spontaneous locomotor activity in animals fed the ω3-supplemented diet corroborates the potential reduced level of anxiety, a parameter that could facilitate the habituation process, a simple form of learning [3]. Our finding that tuna oil-supplemented mouse lemurs exhibited both lower anxiety in the open field task and better cognitive performances in the Barnes maze also suggests that the two outcomes may be directly linked. Indeed, the tendency to higher exploration in the Barnes maze of the tuna oil supplemented animals (higher number of visits and lower time spent to find the right exit) may be issued from the lowering of anxiety, resulting in a higher score in comparison with more anxious and less exploring animals (trend) of the control group. The difference of performance between the two groups might depend more on their anxiety level than on their intrinsic cognitive capacity. Similar findings have been made in rodents which exhibited increased level of anxiety upon chronic ω3 PUFA dietary deficiency [6], [4], and decreased anxiety upon DHA supplementation [4]. Therefore, it is possible that lowering of anxiety underlies better cognitive performances of mouse lemurs raised on the tuna oil-enriched diet.

Despite the potential link between anxiety and cognitive performances, we can not exclude that the better performances in the Barnes maze could result from a direct improvement of spatial reference memory due to long-chain ω3 PUFA supplementation. Indeed, it has been shown that long chain ω3 PUFA play a major role in brain functions and that they are implicated in numerous physiological mechanisms (reviewed in [32]). Studies in rodents have notably demonstrated that ω3 PUFA-deficient animals exhibit severe cognitive impairments that have been linked to changes in neurotransmission processes [33]. The neurotransmission process being highly dependent on energy supply, the effects of ω3 fatty acids could occur at the primary level of glucose utilization by neuronal cells. We have previously shown that ω3 PUFA deficiency [34], [35] and supplementation [36], [37] play a role in brain glucose utilization in rats, which could underlie behavioral changes with ω3 PUFA dietary treatments. On the other hand, Tsukada et al. [14] have shown that a short term dietary DHA supplementation increased the cerebral blood flow of aged monkeys, a parameter that is susceptible to impact on brain glucose utilization and thus on neuronal activation. On the basis of these observations, we may hypothesize that long-chain ω3 PUFA improve cognitive performances in non-human primates through the enhancing of brain glucose utilization.

The ω3-supplemented animals also exhibited lower levels of night spontaneous locomotor activity, which was not accompanied by significant change of motor performances (no change in the performances in the rotating rod task). In agreement with the present data, lowered locomotor activity has been reported in fish oil-supplemented rats [13]. The present study is the first to evidence that a long-chain ω3 PUFA supplementation lowers locomotor activity in non-human primates. In humans, low dietary ω3 PUFA and low tissue levels of DHA are associated with neurodevelopmental disorders, including attention deficit hyperactivity disorder (ADHD) (reviewed in [15]). Locomotor activity and ω3 PUFA may be linked through changes in monoamine neurotransmitters systems. Several studies have reported that dysregulations of the dopamine system occur in ω3 deficient rats [38], [13] and in ADHD in humans and animals [39]. One study performed in Syrian hamsters (a nocturnal species) reported that ω3 PUFA-deficiency caused a chronic locomotor hyperactivity which was associated with disturbance in melatonin rhythm and with hyperdopaminergia [12]. It was proposed that lowering of ω3 PUFA alters the melatonin rhythm, weakens endogenous functioning of the circadian clock, resulting in sleep disturbances as it is observed in ADHD patients [12]. However, it is noteworthy that in our study, only night locomotor activity was significantly changed (active period) without any change in day locomotor activity (resting period), suggesting that changes occurring in ω3-supplemented animals do not involve sleep and/or melatonin-related processes. To date, attempts of ω3 PUFA supplementation in order to treat or to lower ADHD symptoms in children have not been or have been only partly successful [40], [41]. Our present finding that ω3 PUFA supplementation lowers the spontaneous locomotor activity in mouse lemurs renders this species an adequate primate model to investigate the role of long-chain ω3 PUFA in the etiology and/or treatment of hyperactivity disorders.

Our study evidences that dietary ω3 fatty acids positively impact on anxiety and cognitive performances in adult mouse lemurs, a non-human primate. The observation of decreased anxiety with ω3 PUFA supplementation is of particular interest in the context of human health. Indeed, even if it exists significant evidence supporting the potential anxiolytic effect of ω3 PUFA in rodents, there is a lack of studies to demonstrate it, more particularly in primates, *a fortiori* in humans [16]. The present observations are also very encouraging in the context of aging-associated cognitive decline, in which handling of dietary ω3 fatty acids could offer an efficient strategy for sustaining cognitive functions. Further studies are in progress to characterize the impact of long chain ω3 PUFA on behavioral and cognitive performances in this species and to determine whether the effects on cognitive performances are attributable to direct improvement of neuronal functions and/or to lowering of anxiety.
